# Selection of Candidate Reference Genes for Gene Expression Analysis in Kentucky Bluegrass (*Poa pratensis* L.) under Abiotic Stress

**DOI:** 10.3389/fpls.2017.00193

**Published:** 2017-02-14

**Authors:** Kuiju Niu, Yi Shi, Huiling Ma

**Affiliations:** Key Laboratory of Grassland Ecosystem of Ministry of Education, College of Grassland Science, Gansu Agricultural UniversityLanzhou, China

**Keywords:** Kentucky bluegrass, abiotic stress, reference gene, real-time quantitative PCR, gene expression

## Abstract

Kentucky bluegrass (*Poa pratensis* L.) belong to Gramineae and is widely used in lawns, golf courses, landscapes, and sport fields as a prominent cool-season grass. Gene expression patterns during different stages of plant development can provide clues toward the understanding of its biological functions. The selection and validation of reference genes are the first steps in any real-time quantitative PCR gene expression study. Therefore, suitable reference genes are necessary for obtaining reliable results in real-time quantitative PCR analyses of Kentucky bluegrass. In the present study, 9 candidate reference genes were chosen, and their expression stability in the leaves and roots of Kentucky bluegrass under different stresses (drought, salt, heat, and cold) were evaluated using the GeNorm, NormFinder, BestKeeper, and RefFinder programs. The results showed that the expression stability of the candidate reference genes was dependent on the experimental conditions. The combination of SAM with GAPDH was the most stable in leaves under salt stress and cold stress, while TUB combined with ACT or GAPDH was stable in roots under salt or cold stress, respectively. ACT and SAM maintained stable expression in drought-treated leaves, and GAPDH combined with ACT was stable in drought-treated roots. SAM and TUB exhibited stable expression in heat-treated leaves. ACT and RPL were stable in heat-treated roots. In addition, the expression patterns of PpFEH in response to drought and cold stress were used to confirm the reliability of the selected reference genes, indicating that the use of an inappropriate reference gene as the internal control will cause erroneous results. This work is the first study on the expression stability of reference genes in Kentucky bluegrass and will be particularly useful in the selection of stress-tolerance genes and the identification of the molecular mechanisms conferring stress tolerance in this species.

## Introduction

The study of gene expression is fundamental to understanding signal transduction, metabolic pathways, and development. Northern blotting, DNA microarray hybridization and real-time quantitative polymerase chain reaction (RT-qPCR) are commonly used to determine gene expression in response to abiotic stress (Le et al., 2012). RT-qPCR analysis is the most common technique for studying the expression of genes because it is sensitive, specific, cost-effective, and reproducible, and it has been widely applied to study variation in gene expression in diverse organisms and tissues and under different stress conditions (Hong et al., 2008; Chen et al., 2015; Kanakachari et al., 2016). However, the application of RT-qPCR is often affected by methodological errors, including variations in the quality of RNA, variations in the efficiency of cDNA synthesis, and variations in the efficiency of amplification (Delporte et al., 2015). These errors will cause the quantitation of target gene transcripts to be unreliable. Therefore, RT-qPCR data should be normalized. It has been widely reported that the most effective way to normalize the data is to use appropriate reference genes (Hong et al., 2008; Delporte et al., 2015).

Ideal reference genes would be expressed at a constant level in each cell under different physical conditions and would thus be representative of the cDNA concentration in each sample, but there are often variations in their expression due to different experimental treatments (Bustin et al., 2005; Sgamma et al., 2016). Therefore, it is crucial to select reference genes with relatively constant expression. Common reference genes include the basic components of the cytoskeleton (*ACT, TUA, TUB*) or genes involved in the basic biochemical metabolism of organisms (*GAPDH, EF-1a, UBQ*), as they are stably expressed in some tissues and organs (Huggett et al., 2005; Gutierrez et al., 2008). Recent studies have found that there is also variation in the expression of these genes in different cells and tissues and in different developmental stages and various experimental treatments (Hu et al., 2009; Die et al., 2010). Therefore, an increasing number of studies on the identification and evaluation of the stability of reference genes in many types of plants under different conditions have been reported, including *Saccharum sinensis* (Iskandar et al., 2004), *Brachypodium distachyon* (Hong et al., 2008), *Oryza sativa* (Jain, 2009), *Triticum aestivum* (Paolacci et al., 2009), *Solanum lycopersicum* (Long et al., 2010), *Pisum sativum* (Die et al., 2010), *Hordeum vulgare* (Rapacz et al., 2012), *Lactuca sativa* (Borowski et al., 2014; Sgamma et al., 2016), *Buchloe dactyloides* (Li et al., 2014), *Festuca arundinacea* (Yang et al., 2015), and *Agrostis stolonifera* (Chen et al., 2015).

Kentucky bluegrass (*Poa pratensis* L.) belongs to the *Gramineae* family and is widely used in lawns, golf courses, landscapes, and sports fields as a prominent cool-season grass (Puyang et al., 2015). This grass is often subjected to various abiotic stresses, which result in reduced aesthetic quality and seed yield. Among all types of abiotic stress, drought, salt, heat, and cold stress are the most common detrimental factors affecting its growth in various regions (Huang et al., 2014). The validation of reference genes under various stress conditions is necessary for understanding the molecular mechanisms underlying stress responses and cultivating superior stress-tolerant germplasm through biotechnology breeding programs for Kentucky bluegrass. It is worth noting that no research has been conducted to identify and validate reference genes in Kentucky bluegrass.

In the present study, 9 candidate reference genes, *18S, ACT, EF-1a, GAPDH, RPL, RUBP, SAM, TUA*, and *TUB*, were selected, and the expression stability of these genes was evaluated to validate stable reference genes for use in RT-qPCR analysis of gene expression in leaves and roots of Kentucky bluegrass under drought, salt, heat, and cold stress. In addition, the expression of a target gene (*PpFEH*) related to the treatments evaluated was investigated to demonstrate the effectiveness of the selected reference genes identified during the study.

## Materials and methods

### Preparation of plant materials

Kentucky bluegrass (cv. Midnight II) seeds were soaked overnight in tap water and then washed several times to rinse away the empty seeds floating on the water. The washed seeds were sown in plastic pots filled with sand and maintained in a growth chamber controlled at 25/25°C (day/night) with a daily photoperiod cycle of 14 h and relative humidity of 65%. After 3 weeks of seeding, seedlings were transferred into half-strength Hoagland's nutrient solution (Hoagland and Arnon, 1950). The hydroponic culture period lasted for 4 weeks, and the nutrient solution was refreshed weekly during this period.

### Abiotic stress treatments

Seedlings of uniform size were selected and divided into four groups to impose four abiotic stress conditions. For drought or salt treatment, plants were transferred to nutrient solution containing 25% PEG 6000 with −1.25 MPa osmotic potential or 250 mM NaCl, respectively. Heat stress was imposed in a growth chamber set at 40°C, and cold stress was imposed at 4°C in an incubator. At 0, 2, 4, 8, 16, and 24 h of stress treatment, leaves and roots were harvested and immediately frozen in liquid nitrogen and stored at −80°C for further analysis. Each treatment type and duration was replicated three times in three containers of nutrient solution containing PEG 6000 (drought) or NaCl (salt) or three chambers for the heat or cold treatment.

### Total RNA isolation and first-strand cDNA synthesis

Total RNA was isolated from Kentucky bluegrass leaves and roots using the RNAsimple Total RNA Kit (TIANGEN) according to the kit instructions. The RNA quality and concentration were measured with a spectrophotometer (NanoVue™ plus, Wilmington, DE, USA), and samples with an A_260_/A_280_ ratio within 1.8–2.2 and an A_260_/A_230_ ratio of ~2.0 were retained. The removal of genomic DNA contamination and 1st strand cDNA synthesis were performed using the PrimeScript™ RT reagent Kit with gDNA Eraser (TaKaRa) following the manufacturer's instructions.

### Selection of candidate reference genes and primer design

A total of 9 candidate reference genes (*18S, ACT, EF-1a, GAPDH, RPL, RUBP, SAM, TUA, TUB*) that have been reported to be good potential candidates in previously published papers were selected (Hong et al., 2008; Rapacz et al., 2012; Kanakachari et al., 2016). Although many gene high-throughput sequence approaches have been widely provided on plant genetic research, Kentuchy bluegrass is still a less studied species with these approaches. Raw data of Kentuchy bluegrass transcriptome sequences can be found from Genebank database (Bushman et al., 2016; Gan et al., 2016), while it did no help to identify candidate reference genes with these short reads. Due to the absence of draft genome and EST sequences, we could not complete a blast search using the homologous sequences from Arabidopsis or another species as a query to identify the coding DNA sequences of candidate reference genes in Kentucky bluegrass in the EST database. Therefore, we had to clone a 500–800-bp core sequence of these genes using the known sequences of some closely related species (e.g., *B. distachyon, T. aestivum, O. sativa, H. vulgare*, and *Zea mays*) before this research. The cloned sequences of the candidate reference genes are provided in Supplementary material Data Sheet 1. The coding sequences of the candidate reference genes were used to design primers using Primer3 (http://bioinfo.ut.ee/primer3-0.4.0/) according to the following parameters: Melting temperature (Tm) of 58–62°C, with an ideal Tm of 60°C; GC content of 45–55%, with an ideal content of 50%; an optimum length of 17–23 bp and amplicon lengths of 50–200 bp. Descriptions of the candidate reference genes and primer sequences are shown in Table 1.

**Table 1:** **Description of the candidate reference genes, primer sequences and RT–qPCR amplification efficiencies**.

**Gene symbol**	**Definition**	**Primer sequence (5′–3′) (forward/reverse)**	**Amplicon product**		**RT-qPCR efficiency(E)/%**
			**Length**	**Tm(°C)**	
*18S*	*18S* ribosomal RNA	GAAAGACGAACA*ACT*GCGAAAGC/GGCGGAGTCCTATAAGCAACATC	149	85.0	108.4
*ACT*	Actin7	TGTTGGATTCTGGTGATGGTGTC/AGGATGGCGTGCGGAAGG	73	83.0	97.1
*EF-1a*	Elongation factor-1a	TCCCCTTCGTCCCAATCTCTG/TGCCACCAATCTTGTAGACATCC	177	88.5	102.8
*GAPDH*	Glyceraldehyde 3-phosphate Dehydrogenase	AAGG*ACT*GGAGAGGTGGAAGG/AGTGCTGCTTGGAATGATGTTG	54	84.5	109.1
*RPL*	Ribosomal protein L2	GATTGTTCAGGTCGCTGGTG/CAACAGGTTTCATGGGCACA	127	87.0	107.6
*RUBP*	Ribulose 1,5 bisphosphate	TGTGCTGCCTCTTCATCAACG/GCCGCCCATCCGACCTG	53	87.0	94.2
*SAM*	S-Adenosylmethionine decarboxylase	GCTTCTCTGAGGAGGTTGATGTC/GCTCGGTGGCATAGTAGATGTG	126	87.0	95.3
*TUA*	Tubulin alpha	CCAACCTACACCAACCTCAACAG/GGTTTGATGGTGCTCTGAATGTTG	88	82.5	91.6
*TUB*	Alpha tubulin-2B	*ACT*GATGTGGCGGTCCTTCTC/CTGTTGAGGTTGGTGTAGGTTGG	95	84.9	105.0
*PpFEH*	Fructan exohydrolase	AGGTTGGGCGGGTATCTATG/CAGCCTGCACAGTGTCAATT	182	87.5	93.1

For verifying the cloned sequences, one library of the raw reads (SRR3080330) of Kentucky bluegrass transcriptom was downloaded from NCBI (https://www.ncbi.nlm.nih.gov/), which was submitted with the bioproject PRJNA307470. Trinity (v2.1.1, https://github.com/trinityrnaseq/trinityrnaseq/wiki) was used for transcriptom assembling. The stats of the assembly are provided in Supplementary material Table 1. The cloned sequences of 9 candidate reference genes were blasted with the transcriptom. The result showed that not only single hit can be found in the transcriptom for each reference genes, but one or two similar hits can be found with a more than 90% identity. However, the output of blast results of qpcr primers against the transcriptom can demonstrate that all of the primers can be aligned only on these similar genes, and the amplicon products of each pair of primers in these similar genes are exactly the same with the amplicon in the cloned sequences. The blast results can be found in Supplementary material Table 2. This result demonstrated that the cloned sequences are credible for the following analysis.

**Figure 1:**
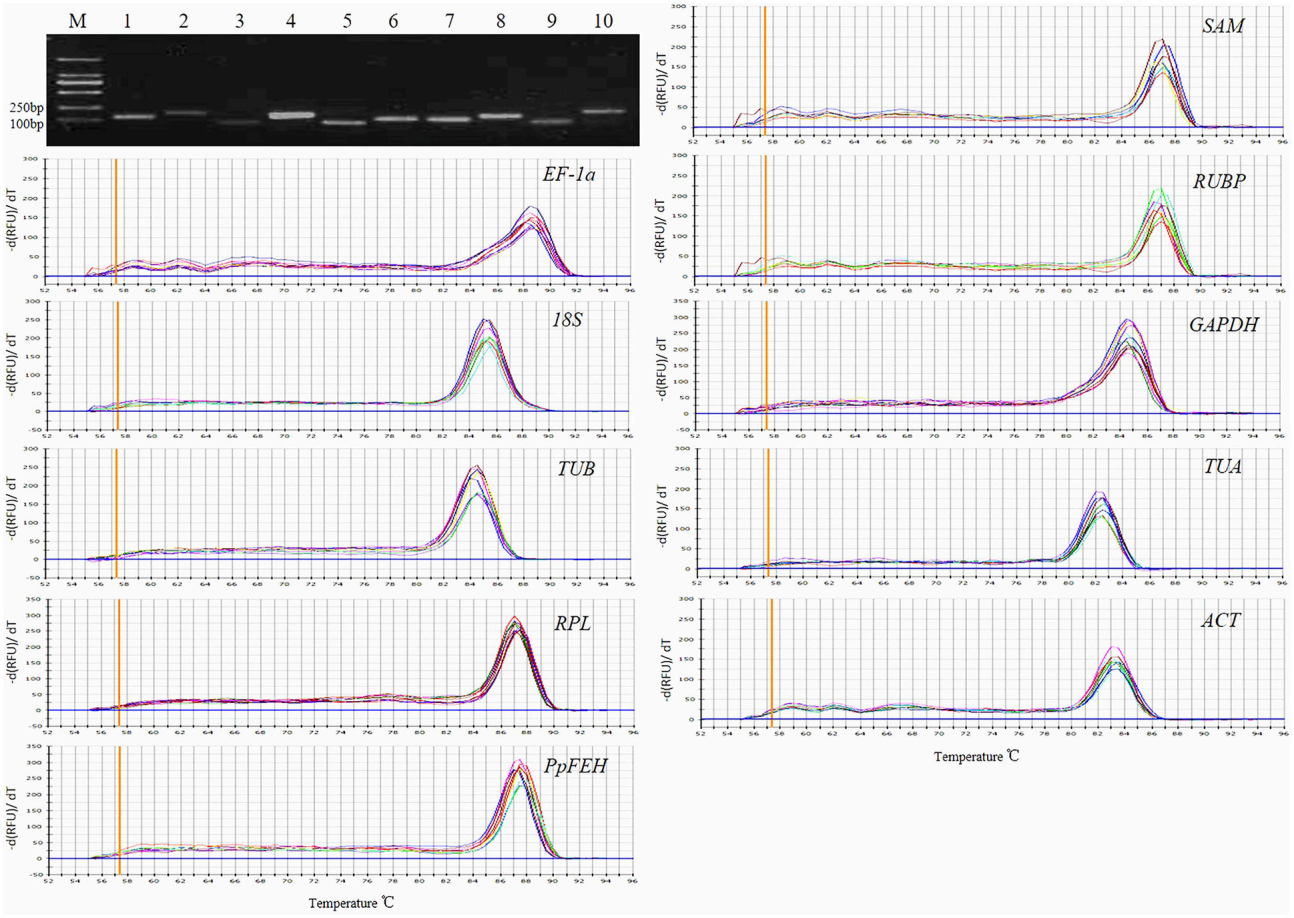
**Primer specificity and amplicon size**. Agarose gel (1.5%) electrophoresis indicated the amplification of a single PCR product of the expected size for 10 genes (lines 1–10: *SAM*, EF-1α, *RUBP, 18S, GAPDH, TUB, TUA, RPL, ACT, PpFEH*). The melting curves for the 10 genes show single peaks. M represents a 2000 bp DNA marker.

The primer specificities were verified by the presence of a single DNA band with the expected size in 1.5% agarose gel electrophoresis and the presence of a single peak in RT-qPCR melting curve assays (Figure 1). RT-qPCR amplification efficiencies for each reference gene were measured using LinRegPCR software (Ruijter et al., 2009). The theoretical optimum value is 100%, indicating that the template is duplicated in an exponential manner, and the acceptable range is usually set between 90 and 110% (Bustin et al., 2009; Sgamma et al., 2016). The RT-qPCR efficiencies for the 9 reference genes and 1 target gene (*PpFEH*) varied from 93.1 to 109.1%, representing acceptable efficiency (Table 1).

### Real-time quantitative PCR analysis

RT-qPCR was conducted with an iQ™ 5 real time PCR machine (Bio-Rad, USA) using SYBR® Premix DimerEraser™ (TaKaRa). Each 20-μL reaction mixture contained 2 μL DNA, 10 μL 2 × SYBR® Premix DimerEraser, 0.6 μL each primer (10 μM) and 6.8 μL ddH_2_O. The amplification conditions were as follows: 95°C for 3 min followed by 40 cycles of 95°C for 10 s and 55°C for 30 s. The melting curve was produced by varying the amplification temperature from 55 to 95°C, with a temperature increase of 0.5°C per cycle. No-template controls were included, and RT-qPCR analysis of each sample was performed in triplicate.

### Stability analysis of reference genes

Four programs, GeNorm (Vandesompele et al., 2002), NormFinder (Andersen et al., 2004), BestKeeper (Pfaffl et al., 2004) and RefFinder (http://fulxie.0fees.us/?type=reference) were used to evaluate the stability of the nine potential reference genes. For GeNorm and NormFinder, the raw quantification cycle (Cq) values were transformed into relative quantities (*Q*-value) using the formula Q = 2^−ΔCq^, in which ΔCq = each corresponding Cq value—minimum Cq value (Yang et al., 2015). The *Q*-value was then uploaded into the GeNorm applet to calculate the expression stability measurement (*M*-value) based on the average of the pairwise variation for a candidate reference gene with all other genes tested. NormFinder calculates the stability value using an ANOVA-based model to consider the intra- and inter-group variation of the candidate reference genes, with the lowest value representing the highest stability. For BestKeeper, the raw Cq values were used to calculate the coefficient of variance (CV) and the standard deviation (*SD*), with the lowest CV representing the highest stability. RefFinder integrated the data from GeNorm (*M*-values), NormFinder (stability values), BestKeeper (CV and *SD*), and the delta Cq values and then generated the comprehensive ranking.

### Validation of reference gene stability

The previous studies showed that fructan exohydrolase (FEH) transcriptional factors are responsive to abiotic stresses (Rao et al., 2011; Xu et al., 2015). *PpFEH* (accession number GQ383670.1) was identified from the Kentucky bluegrass nucleotide database in GenBank. To confirm the reliability of the selected reference gene, the relative expression profiles of FEH in samples of Kentucky bluegrass leaves under drought stress and roots under cold stress were measured and normalized to the most stable and the least stable reference genes identified by RefFinder. The 2^−ΔΔCq^ method was used to calculate the relative expression data. Three technical replicates were performed for each biological sample.

## Results

### Expression levels and variations in candidate reference genes

The quantification cycle (Cq) value reflected the mRNA transcript level. The Cq value produced from the RT-qPCR analysis was used to detect the expression levels of the 9 candidate reference genes. The expression level of each candidate reference gene is presented in Table 2. The Cq values of all the candidate reference genes under different treatments ranged from 10.5 to 33.2. *18S* had the highest level of expression, with a mean Cq of 11.8, and *EF-1a* exhibited the lowest expression level, with a mean Cq of 26.1 (Table 2). The coefficients of variation (CV) show the degree of variation in the observed values within a sample (lower values mean lower variability). Throughout all the treatments, *18S* showed the lowest variability with a CV value of 5.7%, while *RUBP* was the most variable (CV, 23.4%).

**Table 2:** **Expression levels of the 9 candidate reference genes**.

**Treatment^a^**	**TUA**	**TUB**	**RUBP**	**GAPDH**	**EF-1a**	**SAM**	**ACT**	**RPL**	**18S**
DL	24.5	25.3	19.8	19.6	24.8	20.1	24.6	26.2	12.4
DR	23.1	25.0	29.1	21.5	27	22.7	27.0	25.4	11.6
SL	26.6	27.7	22.1	22.7	26.5	23.0	26.6	27.5	12.5
SR	22.7	23.0	31.3	21.1	24.1	22.3	25.6	23.6	10.5
HL	23.5	24.2	18.4	18.6	20.8	20.2	21.6	21.6	11.1
HR	25.1	25.2	31.5	21.8	26.1	24.2	27.7	26.5	12.1
CL	29.0	29.2	21.1	25.1	29.4	23.9	25.3	28.3	11.7
CR	26.4	27.9	33.2	24.1	30.1	26.6	28.2	27.6	12.0
AV^b^	25.1	25.9	25.8	21.8	26.1	22.9	25.8	25.8	11.8
*SD*^c^	2.13	2.11	6.03	2.16	2.97	2.14	2.10	2.26	0.68
CV (%) ^d^	8.5	8.1	23.4	9.9	11.4	9.3	8.1	8.7	5.7

a *DL and DR, drought-treated leaves and roots, respectively; SL and SR, salt-treated leaves and roots, respectively; HL and HR, heat-treated leaves and roots, respectively; CL and CR, cold-treated leaves and roots, respectively; the same abbreviations are used in the table*.

b *AV, average value*.

c *SD, Standard Deviation*.

d *CV, coefficient of variation*.

### Stability of candidate reference genes

#### Genorm analysis

**Figure 2:**
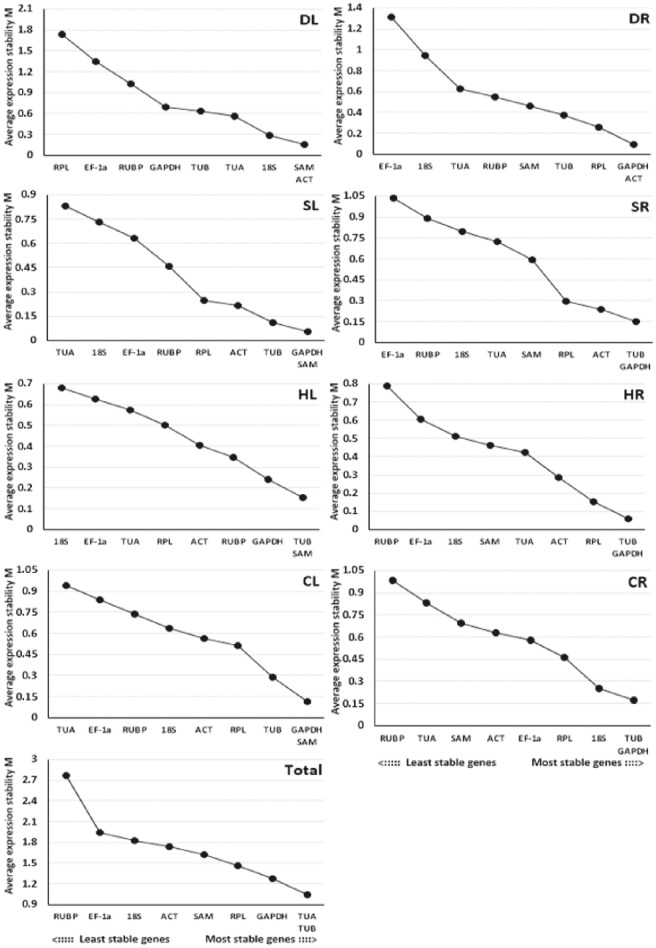
**Average expression stability values (M) of the 9 candidate reference genes assayed with GeNorm software**. The most stable genes on the right and the least stable genes are on the left. DL and DR: Drought-treated leaves and roots, respectively; SL and SR: Salt-treated leaves and roots, respectively; HL and HR: Heat-treated leaves and roots, respectively; CL and CR: Cold-treated leaves and roots, respectively; Total: Pooled samples from all treatments.

The GeNorm program was used to evaluate the stability of the 9 candidate reference genes using the *M*-values, which were defined as the mean variation of a gene relative to all others. In GeNorm, the threshold for eliminating a gene as stable was set as *M* < 1.5, with lower *M*-values indicating higher stability (Vandesompele et al., 2002). Based on this criterion, the results showed that *TUB* and *GAPDH*, which had the same values, were the most stable reference genes for salt-treated roots (SR) or heat-treated and cold-treated roots (HR and CR), while *GAPDH* and *SAM* were the most stable reference genes for salt-treated leaves (SL) or cold-treated leaves (CL), *SAM* and *ACT* were the most stable reference genes for drought-treated leaves (DL), *GAPDH* and *ACT* were the most stable reference genes for drought-treated roots (DR), *TUB* and *SAM* were the most stable reference genes for heat-treated leaves (HL), and *TUA* and *TUB* were the most stable reference genes for pooled samples that included all treatments (Total) (Figure 2). *EF-1a, RUBP*, and *18S* were determined to be the least stable reference genes in most samples.

**Figure 3:**
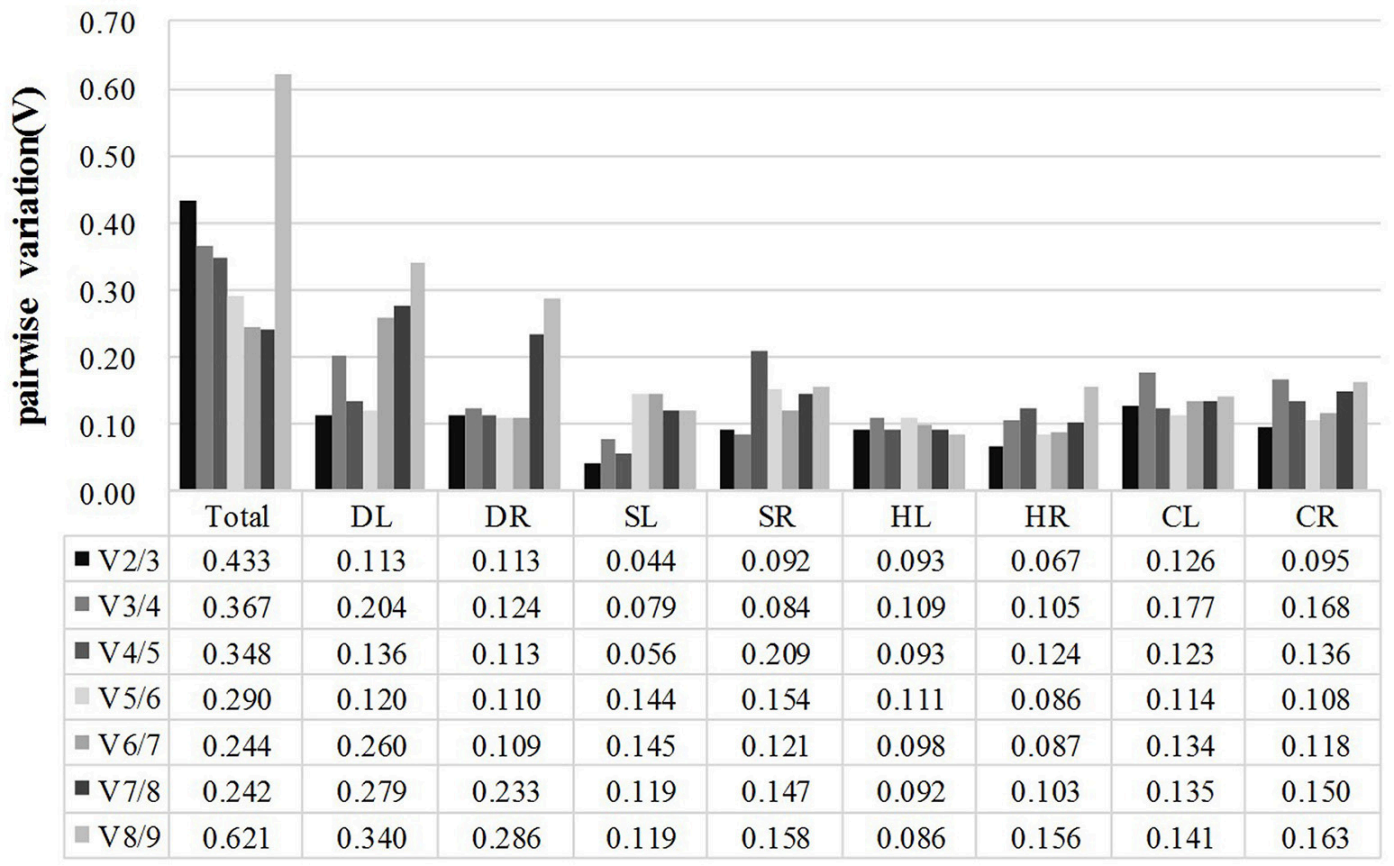
**Pairwise variation (V) of candidate reference genes, as calculated by GeNorm software**. V_*n*_/V_*n*+1_ values were used to determine the optimal number of reference genes. A cut-off of 0.15 (V_*n*_ value) is usually applied. DL and DR: Drought-treated leaves and roots, respectively; SL and SR: Salt-treated leaves and roots, respectively; HL and HR: Heat-treated leaves and roots, respectively; CL and CR: Cold-treated leaves and roots, respectively; Total: Pooled samples from all treatments.

In addition, GeNorm software also provides information on the optimum number of reference genes to be used in the experiment based on the pairwise variation between ranked genes (V_n_/V_n+1_). A cut-off of 0.15 (V_n_ value) is usually applied (Vandesompele et al., 2002). The difference in V_n_ values of V2/3 and V3/4 was noted in this work. The V2/3 values for DL (0.113), DR (0.113), SL (0.044), SR (0.092), HL (0.093), HR (0.067), CL (0.126), and CR (0.095) samples were lower than 0.15 (Figure 3), which indicated that two reference genes would be sufficient for the accurate normalization of these samples. However, a cut-off value of 1.5 should not be considered a rigorous standard, and several reports have found higher cut-off values of V_n_/V_n+1_(Silveira et al., 2009; Chen et al., 2014; Yang et al., 2015). Our data showed a slight variation between V3/4 (0.367) and V4/5 (0.348) in Total samples (pooled samples), suggesting that three genes (*TUA, TUB, GAPDH*) may be necessary for the efficient normalization of all the samples.

#### Normfinder analysis

The stability values of the 9 candidate reference genes were calculated using NormFinder software, with lower values indicating higher stability (Table 3). The three most stable reference genes for all samples (Total) were *SAM* (0.269), *ACT* (0.489), and *GAPDH* (0.499) according to the NormFinder analysis. *SAM* and *GAPDH* were the most stable genes in the SL and CL samples, while *GAPDH* was the lowest stable reference gene in the DL samples. *ACT* was the most stable reference gene in the DL and SR samples. HR and *EF-1a* were each ranked first for their stability in the HR or CR samples, respectively, and exhibited lower stability in the other samples. Consistent with the GeNorm data, *SAM* and *TUB* were the two most stable genes in the HL samples, and *SAM* and *GAPDH* were the most stable genes in the SL and CL samples. However, the stability ranking of the candidate reference genes generated with NormFinder was slightly different from that of GeNorm in most of the samples. For example, *ACT* and *RPL* were identified as the most stable reference genes in the SR and HR samples in the NormFinder analysis, while their stability rankings were third and fourth with the GeNorm analysis, respectively.

**Table 3:** **Stability analysis of candidate reference genes, as assayed with NormFinder software**.

**Rank**	Total^*^	**DL**	**DR**	**SL**	**SR**	**HL**	**HR**	**CL**	**CR**
1	*SAM* (0.269)	*ACT* (0.053)	*TUB* (0.122)	*SAM* (0.137)	*ACT* (0.069)	*SAM* (0.155)	*RPL* (0.074)	*SAM* (0.184)	*EF-1a* (0.197)
2	*ACT* (0.489)	*TUA* (0.125)	*SAM* (0.122)	*GAPDH* (0.147)	*RPL* (0.143)	*TUB* (0.191)	*ACT* (0.111)	*GAPDH* (0.213)	*GAPDH* (0.284)
3	*GAPDH* (0.499)	*TUB* (0.125)	*ACT* (0.359)	*TUB* (0.198)	*TUB* (0.167)	*ACT* (0.257)	*TUB* (0.176)	*RPL* (0.234)	*ACT* (0.297)
4	*RPL* (1.001)	*SAM* (0.146)	*GAPDH* (0.395)	*RPL* (0.339)	*GAPDH* (0.274)	*RUBP* (0.318)	*GAPDH* (0.218)	*TUB* (0.261)	*TUB* (0.323)
5	*TUB* (1.099)	*18S* (0.195)	*TUA* (0.535)	*ACT* (0.395)	*18S* (0.614)	*TUA* (0.343)	*SAM* (0.317)	*ACT* (0.307)	*RPL* (0.409)
6	*EF-1a* (1.173)	*GAPDH* (0.353)	*RUBP* (0.666)	*EF-1a* (0.544)	*TUA* (0.688)	*RPL* (0.362)	*TUA* (0.348)	*18S* (0.504)	*18S* (0.469)
7	*18S* (1.291)	*RUBP* (1.378)	*RPL* (0.666)	*18S* (0.566)	*SAM* (0.694)	*GAPDH* (0.406)	*18S* (0.497)	*RUBP* (0.614)	*TUA* (0.683)
8	*TUA* (1.318)	*EF-1a* (1.665)	*18S* (1.041)	*RUBP* (0.621)	*RUBP* (0.721)	*EF-1a* (0.497)	*EF-1a* (0.583)	*EF-1a* (0.826)	*SAM* (0.711)
9	*RUBP* (3.861)	*RPL* (2.104)	*EF-1a* (1.774)	*TUA* (0.727)	*EF-1a* (0.969)	*18S* (0.524)	*RUBP* (0.963)	*TUA* (0.865)	*RUBP* (1.006)

* *Total: Pooled samples from all treatments. The numbers in parentheses are stability values*.

#### BestKeeper analysis

The expression stability of the 9 candidate reference genes was also evaluated based on the Cq values using the BestKeeper program. In addition, the coefficient of variation (CV) and standard deviation (*SD*) of all the candidate reference genes were calculated. Data with a *SD* < 1 were considered to have acceptable ranges of variation, with lower CV and *SD* indicating higher stability (Migocka and Papierniak, 2010). Table 4 shows the results of the BestKeeper analysis. In the present study, *SAM* was the most stable gene for expression normalization in the DL and HR samples, *GAPDH* was the most stable in the DR and SL samples, *TUA* was the most stable in the HL and CL samples, and *ACT* was the most stable gene in CR and all samples (Total). *18S* was the most stable gene in the SR samples, but exhibited the lowest ranking for the DR, SL, and CR samples. *EF-1a* was ranked fourth in the HR and CR samples and showed the lowest stability in the SR samples. *RUBP* was ranked as the least stably expressed gene in most of the samples, including DL, HL, HR, CL, and Total. Similarly, the stability ranking of the candidate reference genes generated with BestKeeper was different from that of GeNorm and NormFinder for most of the samples.

**Table 4:** **Stability analysis of candidate reference genes, as assayed with BestKeeper software**.

**Rank**	**Total**	**DL**	**DR**	**SL**	**SR**	**HL**	**HR**	**CL**	**CR**
1	*ACT*	*SAM*	*GAPDH*	*GAPDH*	*18S*	*TUA*	*SAM*	*TUA*	*ACT*
CV ±*SD*	6.41 ± 1.66	0.22 ± 0.04	0.31 ± 0.07	0.29 ± 0.07	0.63 ± 0.07	1.61 ± 0.38	0.55 ± 0.13	1.53 ± 0.44	1.26 ± 0.36
2	*TUB*	*ACT*	*ACT*	*SAM*	*TUA*	*18S*	*ACT*	*RPL*	*TUA*
CV ±*SD*	6.7 ± 1.74	0.36 ± 0.09	0.49 ± 0.13	0.39 ± 0.09	1.76 ± 0.4	1.8 ± 0.2	1.12 ± 0.31	1.65 ± 0.47	1.77 ± 0.47
3	*TUA*	*18S*	*RPL*	*RPL*	*SAM*	*TUB*	*TUA*	*18S*	*RPL*
CV ±*SD*	6.81 ± 1.71	1.79 ± 0.22	0.61 ± 0.16	0.57 ± 0.16	2.99 ± 0.67	2.85 ± 0.69	1.24 ± 0.31	1.89 ± 0.22	1.93 ± 0.53
4	*SAM*	*TUA*	*TUB*	*TUB*	*ACT*	*SAM*	*EF-1a*	*ACT*	*EF-1a*
CV ±*SD*	6.95 ± 1.59	2.45 ± 0.60	1.51 ± 0.38	0.64 ± 0.18	3.22 ± 0.82	2.86 ± 0.58	1.45 ± 0.38	2.2 ± 0.56	2.21 ± 0.67
5	*18S*	*TUB*	*RUBP*	*ACT*	*RPL*	*EF-1a*	*RPL*	*GAPDH*	*RUBP*
CV ±*SD*	7.09 ± 0.83	2.64 ± 0.67	1.68 ± 0.49	0.67 ± 0.18	3.49 ± 0.82	3.53 ± 0.73	2.09 ± 0.56	2.66 ± 0.67	2.94 ± 0.98
6	*RPL*	*GAPDH*	*TUA*	*RUBP*	*TUB*	*GAPDH*	*18S*	*TUB*	*TUB*
CV ±*SD*	7.34 ± 1.90	3.06 ± 0.60	2.21 ± 0.51	2.62 ± 0.58	4.16 ± 0.96	3.94 ± 0.73	2.2 ± 0.27	2.74 ± 0.8	3.27 ± 0.91
7	*GAPDH*	*RPL*	*SAM*	*EF-1a*	*RUBP*	*RPL*	*TUB*	*SAM*	*GAPDH*
CV ±*SD*	8.06 ± 1.76	6.62 ± 1.73	2.25 ± 0.51	2.85 ± 0.76	4.47 ± 1.40	4.01 ± 0.87	2.47 ± 0.62	2.98 ± 0.71	4.06 ± 0.98
8	*EF-1a*	*EF-1a*	*EF-1a*	*TUA*	*GAPDH*	*ACT*	*GAPDH*	*EF-1a*	*SAM*
CV ±*SD*	9.69 ± 2.53	7.7 ± 1.91	7.75 ± 2.09	3.18 ± 0.84	4.94 ± 1.04	4.32 ± 0.93	2.96 ± 0.64	4.62 ± 1.36	5.26 ± 1.40
9	*RUBP*	*RUBP*	*18S*	*18S*	*EF-1a*	*RUBP*	*RUBP*	*RUBP*	*18S*
CV ±*SD*	21.24 ± 5.48	8.08 ± 1.60	9.29 ± 1.11	6.6 ± 0.82	5.17 ± 1.24	5.2 ± 0.96	3.88 ± 1.22	5.69 ± 1.20	9.6 ± 1.16

#### RefFinder analysis

The comprehensive rankings of the candidate reference genes shown in Table 5 were determined with the RefFinder program (http://fulxie.0fees.us/?type=reference), which integrates different analysis programs, including GeNorm, NormFinder, BestKeeper and the delta Cq method (Chen et al., 2014, 2015; Yang et al., 2015). *SAM, GAPDH*, and *TUB* were found to be the three most stable reference genes in Total samples. *SAM* and *GAPDH* were suggested to be the most suitable combination for the SL and CL samples. *ACT* combined with *SAM, GAPDH, TUB*, or *RPL* were the most stable reference genes in the DL, DR, SR, and HR samples. The most suitable combination for the HL and CR samples was *TUB* together with *SAM* or *GAPDH*. *EF-1a* or *RUBP* was the least stable reference gene under the majority of the four stress conditions.

**Table 5:** **The most stable and least stable combinations of reference genes, as determined with RefFinder analysis**.

**Experimental treatments**																	
**Total**		**DL**		**DR**		**SL**		**SR**		**HL**		**HR**		**CL**		**CR**	
**Most**	**Least**	**Most**	**Least**	**Most**	**Least**	**Most**	**Least**	**Most**	**Least**	**Most**	**Least**	**Most**	**Least**	**Most**	**Least**	**Most**	**Least**
*SAM*	*RUBP*	*ACT*	*RPL*	*GAPDH*	*EF-1a*	*SAM*	*TUA*	*ACT*	*EF-1a*	*SAM*	*EF-1a*	*ACT*	*RUBP*	*SAM*	*EF-1a*	*GAPDH*	*RUBP*
*GAPDH*		*SAM*		*ACT*		*GAPDH*		*TUB*		*TUB*		*RPL*		*GAPDH*		*TUB*	
*TUB*																	

### Validation of selected reference genes for the expression of target gene, PpFEH

**Figure 4:**
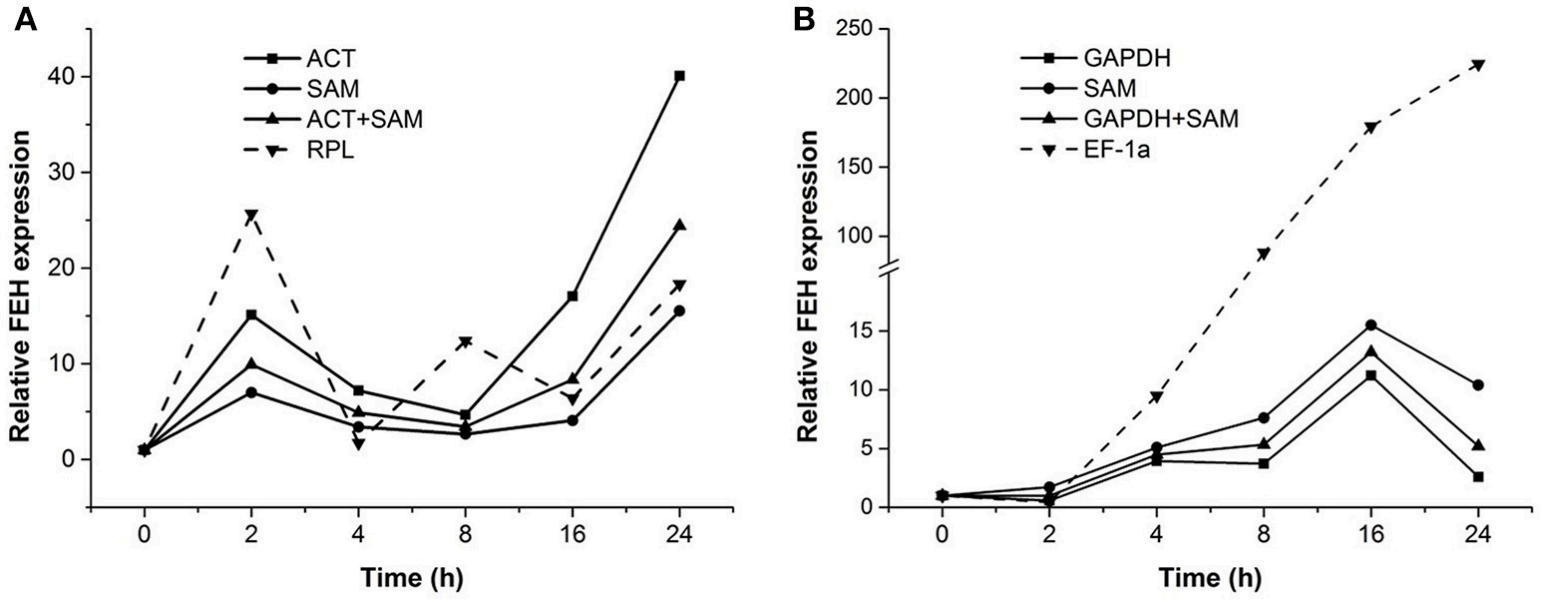
**Relative expression of FEH 0, 2, 4, 8, 16, and 24 h following stress treatment using the selected reference genes for normalization**. **(A)** The expression of FEH in drought-treated leaves was normalized using the two most stable genes (*ACT* and *SAM*) and the least stable gene (*RPL*). **(B)** The expression of FEH in cold-treated roots was normalized using the two most stable genes (*GAPDH* and *SAM*) and the least stable gene (*EF-1a*).

To confirm the stability of reference genes determined to be most or least stable through the analyses described above, the expression pattern of a target gene, *PpFEH*, was examined in response to drought and cold stress in DL and CR samples (Figure 4). The two most stable references genes (*ACT* and *SAM* for DL samples, *GAPDH* and *SAM* for CR samples) and the least stable reference gene (*RPL* for DL samples, EF-1α for CR samples) were used in the validation test. Using *ACT* or *SAM* alone or *ACT* combined with *SAM* as the reference gene, *PpFEH* expression in the leaves of Kentucky bluegrass showed slight fluctuations before 16 h and then significantly increased after 24 h of drought stress, while the maximum expression level occurred at 2 h when *RPL* was used as a reference during drought stress (Figure 4A). In response to cold stress, *PpFEH* expression in the roots of Kentucky bluegrass was clearly up-regulated to the highest level at 16 h of treatment and then declined when *GAPDH* or *SAM* alone or *GAPDH* combined with *SAM* was used as a reference, while the expression of *PpFEH* continuously increased during drought stress and was markedly higher when *TUB* was used as a reference for normalization than when the combination of *GAPDH* and *SAM* was used as a reference after 4 h (Figure 4B).

## Discussion

It is very convenient to obtain the sequences of reference genes in the EST database with a blast search using the Arabidopsis homology sequence or other species as a query, and this is the main method for selecting reference genes for many plant species (Rapacz et al., 2012; Borowski et al., 2014; Kanakachari et al., 2016; Sgamma et al., 2016). In recent years, studies aimed at identifying suitable reference genes as internal controls for RT-qPCR analysis have been reported in many turfgrass species, such as *Lolium perenne* (Lee et al., 2010), bermudagrass (Chen et al., 2014), buffalo grass (Li et al., 2014), tall fescue (Yang et al., 2015), and creeping bentgrass (Chen et al., 2015). However, experiments aimed at selecting stable reference genes for Kentucky bluegrass have not yet been conducted, which might be due to the fact that nothing is present to be searched in the Kentucky bluegrass GenBank EST database. For that reason, we cloned 9 frequently used reference genes before this research.

GeNorm, NormFinder, and BestKeeper are three programs based on statistical analysis that are commonly used by researchers (Yuan et al., 2012). The operating principle of NormFinder is similar to that of the GeNorm program, but the latter can select suitable reference gene combinations and the optimal number of reference genes. In contrast to GeNorm and NormFinder, both the stability of reference genes and the stability of target genes can be analyzed by the BestKeeper program. In the present paper, the rankings created by GeNorm and NormFinder were similar, while the ranking obtained by the BestKeeper program was almost always different. A previous report revealed a similar difference between BestKeeper and other methods (Rapacz et al., 2012). RefFinder, which is regarded as a comprehensive evaluation program and provides information using data from GeNorm, NormFinder, and BestKeeper, selects a stable single gene or gene combination as the internal control (Chen et al., 2014). For this reason, four programs (GeNorm, NormFinder, BestKeeper, and RefFinder) were used to select the stable genes for Kentucky bluegrass. In previous studies, similar methods have been used in many species, such as *A. stolonifera* (Chen et al., 2015), *Cichorium intybus* (Delporte et al., 2015), and *Cynodon dactylon* (Chen et al., 2014).

In this study, 9 genes that have been commonly used as the candidate reference gene in many species were evaluated. According to previous studies on the selection of plant reference genes, the expression level of a reference gene might not be constant across various species (Die et al., 2010; Rapacz et al., 2012; Chen et al., 2015; Sgamma et al., 2016). *GAPDH* exhibited good stability in Kentucky bluegrass roots under drought and cold treatments in this study but was the least stable reference gene for *T. aestivum* (Long et al., 2010). Our study demonstrated that *EF-1a* was the least stable reference gene in roots of Kentucky bluegrass exposed to drought and salt stresses, as well as leaves under cold stress. However, *EF-1a* revealed good *stability* in soybean (*Glycine max*) under drought and salt stresses (Ma et al., 2013), in *Populus euphratica* under cold stress (Wang et al., 2014), and in *Caragana korshinskii* under heat stress (Yang et al., 2014).

The expression of a reference gene can be different in the same species in response to various treatments. In this study, *RPL* was the least stable reference gene in Kentucky bluegrass leaves exposed to drought stress but showed good stability in heat-treated roots. In a previous study, *ACT* was the least stable reference gene in leaves and roots under cold treatment, while good stability was detected in salt-treated leaves of creeping bentgrass (*A. stolonifera*) (Chen et al., 2015). In addition, the stability of individual members of reference gene families can be diverse, which indicates that the stability of other members cannot be determined based on one member's stability. *TUA* and *TUB* of the tubulin gene family (structural proteins in the cytoskeleton) are widely used as reference genes. In our study, the expression stability of *TUB* expression was commonly higher than *TUA* in the GeNorm and NormFinder analyses. Genes in the actin family, which includes *ACT*2/7, *ACT*8, and *ACT*11, have also been used as reference genes. Hu et al. found that the expression stability of *ACT*11 was much higher than that of *ACT*2/7 in soybean (Hu et al., 2009).

The reliability of reference genes exhibiting the highest or lowest stability was further validated by determining the expression patterns of a target gene, *PpFEH*, in Kentucky bluegrass in response to drought and cold stress. Our results showed that the expression of *PpFEH* exhibited a clear pattern in response to drought or cold stress when the combination of *ACT* and *SAM* or *GAPDH* and *SAM* was used as a reference; therefore, these genes could be suitable for the quantification of target gene expression in cold-stressed roots or drought-stressed leaves of Kentucky bluegrass. *PpFEH* exhibited variable expression patterns when *RPL* was used as a reference gene in drought-stressed leaves or when EH-1a was used in cold-stressed roots, suggesting that those genes would be unreliable for RT-qPCR analysis in cold- or drought-stressed Kentucky bluegrass. Similarly, some studies have reported that significant variations in target gene expression levels were detected when unstable reference genes were used as the internal control for RT-qPCR analysis, leading to the misinterpretation of experimental results (Chen et al., 2014, 2015; Wang et al., 2014). Therefore, selecting suitable reference genes is extremely important for the normalization of target gene expression data generated with RT-qPCR.

In summary, in this study, we conducted the first comprehensive analysis of the selection of stable reference genes for RT-qPCR analysis of target gene expression in leaves and roots of Kentucky bluegrass under four different abiotic stresses. The combination of *SAM* with *GAPDH* was suitable for gene quantification in leaves of Kentucky bluegrass under salt and cold stress, while *TUB* combined with *ACT* or *GAPDH* were the two most stable reference genes in roots of Kentucky bluegrass under salt or cold stress, respectively. *ACT* and *SAM* maintained stable expression in drought-treated leaves, and the combination of *GAPDH* and *ACT* served as a stable reference gene in drought-treated roots of Kentucky bluegrass. *SAM* and *TUB* were the two most stable reference genes in heat-treated leaves, and the combination of *ACT* and *RPL* could be used for heat-treated roots of Kentucky bluegrass. The results acquired in this study will improve the accuracy of quantification of target gene expression with RT-qPCR analysis in Kentucky bluegrass under four common abiotic stresses and will facilitate the identification of stress-responsive genes and the molecular mechanisms conferring stress tolerance in this species.

## Author contributions

KN and HM carried out the experimental design. KN performed the experiments and prepared the manuscript and coordinated its revision. YS read and revised the manuscript. All authors provided helpful discussions and approved its final version.

## Funding

The research was supported by National Natural Science Foundation of China (NSFC) (project # 31160482).

### Conflict of interest statement

The authors declare that the research was conducted in the absence of any commercial or financial relationships that could be construed as a potential conflict of interest.

## Abbreviations

*18S*: *18S* ribosomal RNA
*ACT*: Actin7
*EF-1a*: Elongation factor-1a
*GAPDH*: Glyceraldehyde 3-phosphate dehydrogenase
*RPL*: Ribosomal protein L2
*RUBP*: Ribulose 1, 5 bisphosphate
*SAM*: S-Adenosylmethionine decarboxylase
*TUA*: Tubulin alpha
*TUB*: Alpha tubulin-2B
*PpFEH*: Fructan exohydrolase of *Poa pratensis*.
