# Supplementary material for: Selection of Candidate Reference Genes for Gene Expression Analysis in Kentucky Bluegrass (Poa pratensis L.) under Abiotic Stress
Source: Front Plant Sci. 2017 Feb 14;8:193. doi: 10.3389/fpls.2017.00193 (PMC5306334; doi:10.3389/fpls.2017.00193)
Supplement: Supplementary file 1 [file Table1.DOC]

Supplementary Table 1. Summary on assembled transcripts and unigenes of kentucky bluegrass transcriptom

| Total transcripts | 426084 |
| --- | --- |
| Transcripts average length (bp) | 532.96 |
| Total bases of trasncripts (bp) | 227083836 |
| Total of unigenes | 257025 |
| Unigenes average length (bp) | 461.89 |
| Total bases of unigenes (bp) | 118717770 |
| N50 of unigenes (bp) | 506 |
