# Supplementary material for: Selection of Candidate Reference Genes for Gene Expression Analysis in Kentucky Bluegrass (Poa pratensis L.) under Abiotic Stress
Source: Front Plant Sci. 2017 Feb 14;8:193. doi: 10.3389/fpls.2017.00193 (PMC5306334; doi:10.3389/fpls.2017.00193)
Supplement: Supplementary file 2 [file Table2.DOC]

Supplementary Table 2 The blast results of cloned sequences and qpcr primers with the assembled transcriptom

| Query | Length | Qpcr primer (forward/reverse) | Amplicon product length | Hit1 | Hit1 evalue | Hit1 identity | Amplicon product length in hit 1 | Hit2 | Hit2 evalue | Hit2 identity | Amplicon product length in hit 2 |
| --- | --- | --- | --- | --- | --- | --- | --- | --- | --- | --- | --- |
| 18SRNA | 695 | GAAAGACGAACAACTGCGAAAGC/  GGCGGAGTCCTATAAGCAACATC | 149 | TRINITY_DN97600_c0_g16_i1 | 0 | 671/671 | 149 | TRINITY_DN97031_c0_g3_i1 | 0 | 671/671 | 149 |
| Actin | 673 | TGTTGGATTCTGGTGATGGTGTC/  AGGATGGCGTGCGGAAGG | 73 | TRINITY_DN119353_c1_g3_i1 | 0 | 638/640 | 73 | TRINITY_DN117691_c0_g7_i1 | 0 | 637/640 | 73 |
| EF-1a | 523 | TCCCCTTCGTCCCAATCTCTG/  TGCCACCAATCTTGTAGACATCC | 177 | TRINITY_DN121724_c4_g3_i4 | 0 | 371/379 | 177 | TRINITY_DN122008_c4_g18_i2 | 0 | 371/379 | 177 |
| GADPH | 609 | AAGGACTGGAGAGGTGGAAGG/  AGTGCTGCTTGGAATGATGTTG | 54 | TRINITY_DN116042_c2_g7_i1 | 0 | 568/572 | 54 | TRINITY_DN114372_c0_g4_i1 | 0 | 458/463 | 54 |
| RPL | 488 | GATTGTTCAGGTCGCTGGTG/  CAACAGGTTTCATGGGCACA | 127 | TRINITY_DN122342_c0_g1_i2 | 2.00E-79 | 453/488 | 127 | TRINITY_DN122342_c1_g2_i1 | 1.00E-74 | 455/533 | 127 |
| RUBP | 521 | TGTGCTGCCTCTTCATCAACG/  GCCGCCCATCCGACCTG | 53 | TRINITY_DN113503_c12_g11_i3 | 0 | 490/492 | 53 | TRINITY_DN117868_c5_g4_i2 | 0 | 434/435 | 53 |
| SamDC | 715 | GCTTCTCTGAGGAGGTTGATGTC/  GCTCGGTGGCATAGTAGATGTG | 126 | TRINITY_DN122915_c5_g1_i1 | 0 | 692/708 | 126 | TRINITY_DN123244_c6_g1_i6 | 0 | 685/708 | 126 |
| TUA | 650 | CCAACCTACACCAACCTCAACAG/  GGTTTGATGGTGCTCTGAATGTTG | 88 | TRINITY_DN112031_c3_g3_i1 | 0 | 489/497 | 88 |  |  |  |  |
| TUB | 579 | ACTGATGTGGCGGTCCTTCTC/  CTGTTGAGGTTGGTGTAGGTTGG | 95 | TRINITY_DN116807_c0_g5_i4 | 0 | 528/532 | 95 | TRINITY_DN112031_c3_g3_i3 | 0 | 525/529 | 95 |
